# Seminal vesicle protein *ca*CA12 in *Corydoras aeneus* inhibits sperm motility for sperm drinking

**DOI:** 10.1242/jeb.250293

**Published:** 2025-05-15

**Authors:** Junki Yoshida, Maho Yamamoto, Junki Kamiya, Akinaga Kondo, Yukihito Sakaguchi, Nanami Morino, Takako Saito, Masanori Kohda, Satoshi Awata, Ban Sato, Kenji Miyado, Natsuko Kawano

**Affiliations:** ^1^ Department of Life Sciences, School of Agriculture, Meiji University, Tama-ku, Kawasaki, Kanagawa 214-8571, Japan; ^2^ Organization for the Strategic Coordination of Research and Intellectual Properties, Meiji University, Tama-ku, Kawasaki, Kanagawa 214-8571, Japan; ^3^ Department of Applied Life Science, Faculty of Agriculture, Shizuoka University, Shizuoka 422-8529, Japan; ^4^ Department of Biology, Graduate School of Science, Osaka Metropolitan University, Osaka 558-8585, Japan; ^5^ Department of Biology and Geosciences, Graduate School of Science, Osaka City University, Osaka 558-8585, Japan; ^6^ Department of Reproductive Biology, National Research Institute for Child Health and Development, 2-10-1 Okura, Setagaya-ku, Tokyo 157-8535, Japan

**Keywords:** *Corydoras*, Seminal vesicle fluid, Sperm drinking, Carbonic anhydrase, Inhibition of sperm motility

## Abstract

Seminal vesicle (SV) secretions enhance fertilization by regulating sperm motility and fertilization capacity, and by forming plugs that prevent mating with other males. Although SVs are rare in teleosts, certain species, such as *Corydoras* spp., do possess them*.* In *Corydoras* spp. and other species that exhibit sperm drinking or related behaviors, females attach their mouths to the males' genital pore to ingest semen, a reproductive behavior known as sperm drinking. However, the major proteins and functions of seminal vesicle fluid (SVF) in *Corydoras* remain unidentified. This study aimed to identify the SVF proteins in *Corydoras aeneus* and clarify the functions of the identified major SVF proteins. The SVF of this species was found to be highly viscous with a high protein concentration. Sperm motility was strongly suppressed in the presence of the SVF. We identified three SVF proteins – alpha-2-macroglobulin (A2M), carbonic anhydrase 12 (CA12) and lymphocyte antigen 6 (Ly6) – through RNA sequencing (RNA-Seq), LC-MS/MS and amino acid sequencing. Additionally, we found that the identified CA12, termed ‘*ca*CA12,’ was degraded into about 10 kDa and 33 kDa polypeptides containing the CA domain. The 33 kDa polypeptide with the CA domain was found to inhibit sperm motility. The identified SVF proteins, including *ca*CA12, may play a role in keeping sperm in an immotile state until they are close to the female ova, facilitating the remarkable sperm drinking reproductive process observed in *C. aeneus.*

## Teaser

**Summary:** *Corydoras aeneus* seminal vesicle fluid protein CA12 suppresses sperm motility until sperm are close to the female eggs, facilitating the remarkable sperm drinking reproductive process.

## INTRODUCTION

Seminal plasma plays an important role in fertilization, and the secretions, such as sugars, proteins and ions, from male accessory sex glands are known to regulate sperm motility and survival in mammals (Noda and Ikawa, 2019; Owen and Katz, 2005). In particular, seminal vesicle (SV) proteins regulate sperm motility and fertilization (Kawano et al., 2014; Noda et al., 2019; Noda and Ikawa, 2019; Pang et al., 1979; Peitz and Olds-Clarke, 1986). For example, the mouse SV protein SPINK-like (SPLINKL) can inhibit sperm capacitation and enhance sperm motility *in vitro* (Lin et al., 2008), while human seminal plasma motility inhibitor (SPMI) and mouse seminal vesicle autoantigen (SVA) can suppress sperm motility (Huang et al., 1999; Iwamoto and Gagnon, 1988; Iwamoto et al., 1995). Seminal vesicle secretion 2 (SVS2) contributes to sperm viability by protecting sperm from the immune system in the female reproductive tract in mice (Kawano et al., 2014). Additionally, mouse SVS2, SPINKL, Serpin Peptidase Inhibitor, Clade E, Member 2 (SERPINE2) and human Semenogelin 1 (SEMG1) suppress sperm capacitation and acrosome reaction (Kawano and Yoshida, 2007; Lin et al., 2008; Lu et al., 2011; Tseng et al., 2013).

Teleosts have different fertilization modes from mammals. Spermatozoa of teleost species, except for sturgeons, have no acrosomes and bind eggs through micropyles, which are small channels in the chorion, for fertilization. In addition, most teleost species exhibit external fertilization and hence do not develop male accessory reproductive organs (Chowdhury and Joy, 2007). Some teleost species such as sculpin and scorpionfish perform internal fertilization and have characteristic sperm morphology and physiology that differ from those of species with external fertilization (Ito et al., 2022). However, some catfish and gobies that exhibit polygamy and sperm competition possess SVs, similar to mammals (Mazzoldi et al., 2005). SVs of African catfish have been reported to contain more fructose and protein than the testes (Chowdhury and Joy, 2007). Thus, SVs in teleosts have evolved to adapt to diverse fertilization conditions, influenced by factors such as sperm competition and environmental parameters such as osmotic pressure.

**Fig. 1.**
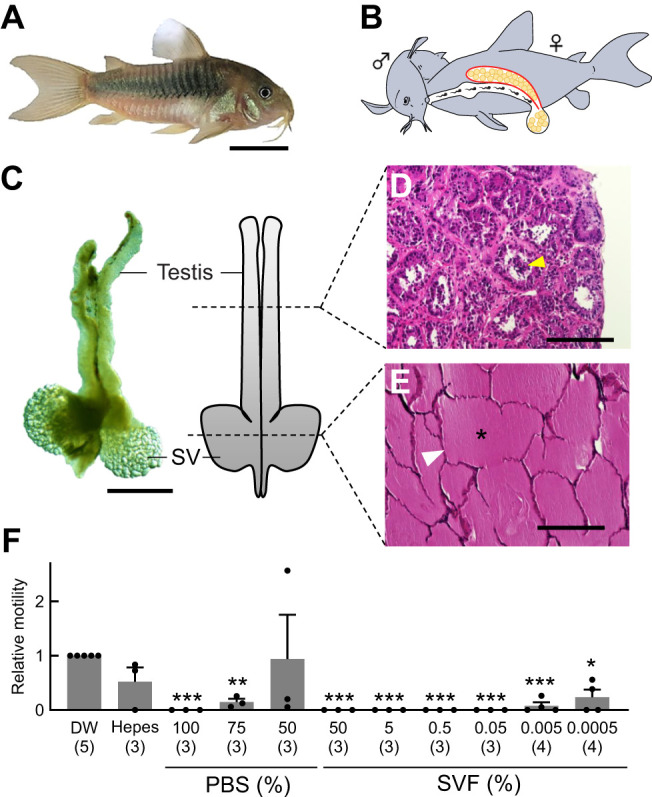
**Male reproductive organs related to reproduction and the function of seminal vesicle fluid (SVF) in *Corydoras aeneus*.** (A) Photograph of an adult male *C. aeneus*. Scale bar: 1 cm. (B) Illustration of sperm drinking, a reproductive behavior in *Corydoras* spp. The white duct in the female is the gastrointestinal tract, and the upper yellow sac is the ovary. In sperm drinking, *Corydoras* sperm migrate through the female gastrointestinal duct and fertilize the eggs at an external place between the pelvic fins. (C) Morphology of the male reproductive organ of *Corydoras*. Photograph (left) and diagram (right) of the male reproductive organ. Dashed lines on the diagram indicate the histological location of the testis and seminal vesicle (SV). Scale bar: 2 mm. (D) Hematoxylin and eosin (HE) staining of the testis. Scale bar: 200 µm. Yellow arrowhead indicates spermatozoa in a cyst. (E) HE staining of the seminal vesicles. Scale bar: 200 µm. White arrowhead indicates secretory epithelium and asterisk indicates eosin-positive SVF inside the epithelium. (F) *Corydoras* sperm motility in Hepes buffer, PBS buffer or SVF at different dilutions. Sperm motility values were normalized to the number of motile spermatozoa in the control condition using distilled water (DW). Independent experiments were performed using spermatozoa collected from different *Corydoras* males. PBS solution was undiluted (100% PBS), or diluted to 75% PBS and 50% PBS with DW; 50% SVF in DW was undiluted (50% SVF), or diluted to 5% SVF, 0.5% SVF, 0.05% SVF, 0.005% SVF and 0.0005% SVF. Data are means+s.d. Numbers in parentheses are number of male *C. aeneus* examined. Unpaired two-tailed Student's *t*-test was conducted (*P<0.05, **P<0.01, ***P<0.001 versus DW).

Members of the genus *Corydoras* (Family: Callichthyidae) (Alexandrou et al., 2011; Liu et al., 2019) possess SVs and have a characteristic fertilization mode known as sperm drinking (Fig. 1A; Kohda et al., 1995; Mazzoldi et al., 2007). In this fertilization mode, the female ingests the spermatozoa by attaching her mouth to the anal region of the male during courtship (Fig. 1B). Thereafter, the sperm pass through the gastrointestinal tract and get released from the general excretory pore of the female. The sperm then reach an external place between the pelvic fins and penetrate the ovulated eggs stored there, which is a form of *in vitro* fertilization (Kohda et al., 2002). In this remarkable reproduction process, *Corydoras* sperm are exposed to two potentially harmful factors compared with the sperm of other teleosts with normal external fertilization: digestive and immune activities in the female gastrointestinal tract. Here, we hypothesized that the secretions from SVs in *Corydoras* protect the sperm from the harmful digestive and immune effects in the female body, as observed in mammals. A previous study has reported that *Corydoras* species exhibiting sperm drinking have well-developed SVs, while species that do not engage in sperm drinking lack specialized accessory organs for SV secretion (Mazzoldi et al., 2007). This observation supports our hypothesis that seminal vesicle fluid (SVF) in *Corydoras* contains functional proteins that may protect and support sperm during and after sperm drinking.

The SVF of *Corydoras* has been reported to contain a high concentration of glycoproteins (Franceschini-Vicentini et al., 2007); however, the specific proteins that are present in the secretions have not been identified yet. In the present study, we identified the SV proteins in *Corydoras aeneus* and determined the function of SVF in its fertilization. The results revealed the presence of three proteins, one of which, carbonic anhydrase 12 (CA12), regulates sperm motility.

**List of abbreviations**

A2M: alpha-2-macroglobulin
CA12: carbonic anhydrase 12
*ca*CA12: *Corydoras aeneus* CA12
DW: distilled water
Ly6: lymphocyte antigen 6
PBS: phosphate-buffered saline
PVDF: polyvinylidene difluoride
RACE: rapid amplification of cDNA ends
RNA-Seq: RNA sequencing
RT: reverse transcription
SV: seminal vesicle
SVF: seminal vesicle fluid
uPAR: urokinase-type plasminogen activator receptor

## MATERIALS AND METHODS

### Ethical statement

This study was approved by the Animal Care Committee of Meiji University (approval numbers: MUIACUC2020-124 and MUIACUC2024-09). In accordance with the institutional guidelines, a minimal number of animals were euthanized in this study, under anesthesia (0.001% FA100; Sumitomo Pharma Animal Health Co., Ltd, Osaka, Japan) or by chilling on ice.

### Fish breeding

Juvenile *Corydoras aeneus* (T. N. Gill 1858) and adult medaka. *Oryzias latipes* ver (Temminck & Schlegel 1846), were purchased from Kamihata Fish Industries Co., Ltd (Himeji, Japan). Fish were maintained in fresh water at 27°C under a 14 h/10 h light/dark photoperiod cycle. *Corydoras aeneus* were fed twice a day with *Chironomidae* larvae and Hikari Crest Corydoras (Kyorin Co., Ltd, Himeji, Japan), and medaka were fed once a day with Hikari Crest Guppy (Kyorin Co., Ltd).

### Histology

The male reproductive organs of *C. aeneus* are shown in Fig. 1C. The testes are paired, narrow and elongated and continue with the sperm ducts that open at the terminal end of the genital papilla. The SV, situated caudal to the testes, is also a paired organ with a large vesicular structure. Gonads were removed from mature males and observed under a stereomicroscope (SMZ800N, Nikon, Tokyo, Japan). The gonad specimens were fixed in 10% (v/v) formalin/phosphate-buffered saline (PBS) for 24 h at room temperature (25–27°C), washed with 1× PBS for 1 h, then soaked in 70% (v/v) ethanol, and finally stored at 4°C. The specimens were dehydrated and soaked in paraffin using an automated fixation and embedding device, RH-12DM (Sakura Finetek, Tokyo, Japan). Paraffin blocks were thinly sliced at 4–6 µm, mounted on glass slides (Muto Chemical, Tokyo, Japan) and incubated overnight at 40°C. The specimens were deparaffinized, hydrophilized and stained with hematoxylin and eosin (HE).

### Sperm motility assay

Mature *C. aeneus* and medaka were euthanized using FA100, and the testes were carefully collected. Using tweezers, a piece of approximately 1 mm of the testis was collected and placed on a glass slide. Next, 5 µl of each solution [distilled water (DW), 10 mmol l^−1^ Hepes/NaOH buffer (pH 7.5) (Hepes), 154 mmol l^−1^ NaCl (saline), PBS, SVF, SV candidates and 50 mmol l^−1^ NaHCO_3_ (HCO_3_^−^)] was dropped onto the piece of testis, and the testis was minced in the solution using tweezers. After treatment, 2 µl of sperm suspension was collected and placed in a disposable chamber (Detect Inc., Panama City, FL, USA) of the Sperm Motility Analysis System (SMAS, Detect Inc.). The sperm motility rate was calculated for sperm swimming with a linear velocity of 5 µm s^−1^ or more. Sperm tracking parameters were set for SMAS analysis (*C. aeneus*, Table S1A; medaka, Table S1B).

SVs collected from mature *C. aeneus* male were crushed with tweezers in an equal volume of 1× PBS. The SVF had a high protein concentration (approximately 13 mg ml^−1^) and was highly viscous. SVF was mixed with an equal volume of 1× PBS to produce 50% SVF. To perform the sperm motility assay, 50% SVF was diluted to appropriate concentrations in DW for *C. aeneus* sperm and in saline for medaka sperm.

### Identification of SV proteins

The collected SVF was separated by sodium dodecyl sulfate-polyacrylamide gel electrophoresis (SDS-PAGE) and transferred onto a polyvinylidene difluoride (PVDF) membrane. After staining the PVDF membrane with Coomassie Brilliant Blue, the candidate bands were excised and subjected to Edman degradation in a PPSQ-33A sequencer (Hokkaido System Science Co., Hokkaido, Japan).

Three candidate proteins separated by SDS-PAGE and stained with Coomassie Brilliant Blue were subjected to nano LC-MS/MS protein identification (Nippon Proteomics Co, Ltd, Miyagi, Japan). Data were searched against the *de novo* assemble data of *C. aeneus* obtained in the present study.

### RNA sequencing of SV tissue

Purified total RNA of the SV collected from mature male *C. aeneus* was paired-end sequenced by Illumina HiSeq 2500 (NGS, Antegral and Macrogen, Japan Inc.), and the read number was 71,266,520. Using short-read data of approximately 100 bp, *de novo* assembly was performed in Galaxy/NAAC (https://galaxy.dna.affrc.go.jp/). Read data were quality controlled using Trimmomatic and assembled using Trinity. The contigs obtained by Trinity were translated into amino acid sequences using Transdecoder. The translated amino acid alignments were subjected to homology searches using BLASTp and domain searches using Pfam to predict the coding regions. A TBLASTN homology search of amino acid alignments, obtained from peptide sequencing or nano LC-MS/MS, was performed based on the data obtained from RNA sequencing (RNA-Seq). The raw RNA-Seq data and *de novo* assembled data obtained in this study can be accessed from the DDBJ database with accession number DRR635078 and with BioProject accession number PRJDB19998.

### Cloning of full-length sequences of protein candidates

To determine the full-length sequence of the candidate *ca*CA12, rapid amplification of cDNA ends (RACE) was performed using a GeneRacer Kit with SuperScript III RT (Thermo Fisher Scientific, Waltham, MA, USA). cDNA was prepared from total RNA of SV tissue through a reverse transcription (RT) reaction using GeneRacer 5′ primer and GeneRacer oligo dT primer. For nested PCR, the 5′ or 3′ end of cDNA was amplified through two PCRs using KOD FX Neo (TOYOBO, Tokyo, Japan) and multiple primer sets (Table S1C). The thermal cycling conditions for the first nested PCR were 94°C for 2 min, followed by 5 cycles of 94°C for 30 s and 72°C for 1 min, 5 cycles of 94°C for 30 s and 70°C for 1 min, and 25 cycles of 94°C for 30 s, 66°C for 30 s, then 68°C for 1 min. The conditions for the second nested PCR were 94°C for 2 min, followed by 25 cycles of 94°C for 30 s, 65°C for 30 s, and 68°C for 1 min. PCR products on gels were cloned using a Zero Blunt^Ⓡ^ TOPO^Ⓡ^ PCR Cloning Kit for Sequencing (Thermo Fisher Scientific) and sequenced using BigDye Terminator v3.1 (Thermo Fisher Scientific) and 3130×l Genetic Analyzer (Thermo Fisher Scientific) to determine transcription start sites and 3′ ends of transcripts. The obtained sequences were checked against the RNA sequence results from NGS.

### Phylogenetic tree analysis and 3D structure prediction from the amino acid sequence of *ca*CA12

Candidate domains were identified for the amino acid sequence from the sequencing results using the conserved domain in NCBI. Alignment and phylogenetic reconstructions were performed using ETE3 3.1.3 (Huerta-Cepas et al., 2016). A maximum-likelihood tree of *ca*CA12 and other known CA proteins was constructed with 100 bootstrap replicates using PhyML v20160115 (Guindon et al., 2010).

The predicted 3D structure of *ca*CA1 was generated from a local copy of Alphafold2 (Jumper et al., 2021) and visualized using PyMOL by Schrödinger (https://pymol.org/2/).

### Recombinant proteins and antibodies

To produce recombinant proteins and corresponding antibodies, the whole molecule and 33 kDa N-terminal sequence of *ca*CA12 were amplified by PCR using primers listed in Table S1C. Each PCR product was subcloned into the plasmid vectors pGEX-6P-1 and pET-24d(+), which were then expressed in *Escherichia coli*. The recombinant proteins were extracted by BugBuster (Merck, Darmstadt, Germany) and purified by Glutathione Sepharose^TM^ 4B (GE Healthcare, Tokyo, Japan) and HIS-Select Nickel Affinity Gel (Sigma-Aldrich, St Louis, MO, USA). The C-terminal repeat sequence of *ca*CA12, TAYPTIQPFAKLPIQP, was synthesized by a peptide synthesis service (GenScript Japan KK, Tokyo, Japan).

Polyclonal antibodies against three candidate proteins – the whole molecule, N-terminus (33 kDa) and C-terminal repeat peptide of CA12 – were developed in BALB/c mice with 50 µg of recombinant protein, following the methods described by Padma et al. (2003). Antibodies in the serum from immunizing mice were purified using HiTrap Protein A HP Columns (GE Healthcare).

### Immunoblotting

SVF and SV tissue extracts were separated using SDS-PAGE and transferred onto a PVDF membrane. The membrane was blocked with 3% (w/v) skimmed milk (Morinaga Milk Industry, Tokyo, Japan) and then incubated with 0.8 µg ml^−1^ mouse IgG against the three types of proteins. The secondary antibody used was HRP-conjugated anti-mouse IgG (Sigma-Aldrich) at 1.1 µg ml^-1^, and signals were developed using SuperSignal^®^ West Dura (Thermo Fisher Scientific).

### Preparing recombinant CA12

Two recombinant proteins – the whole molecule and N-terminus (33 kDa) of *ca*CA12 – were purified using HIS-Select^®^ Nickel Affinity Gel (Sigma-Aldrich). Because imidazole in elution buffers affects sperm motility, Amicon^®^ Ultra-0.5 ml-30K (Sigma-Aldrich) was used to remove the elution buffer.

For the sperm motility assay, three types of recombinant proteins of *ca*CA12 and bovine CA (Sigma-Aldrich) were prepared at a concentration of 41 µmol l^−1^, which is the physiological concentration of *ca*CA12 in the SVF.

## RESULTS

### Histology of the male reproductive tract in *C. aeneus*

In Fig. 1C, the dotted lines indicate where the testis and SV were sectioned transversally at 4–6 µm. HE staining of the testis section revealed small cyst structures surrounded by eosin-positive cells, with very few sperm inside the cyst (Fig. 1D). The SVs were lined by a simple epithelium; their lumen was filled with an eosinophilic fluid and did not contain sperm cells (Fig. 1E).

### Effect of SVF on sperm motility

To determine the effect of SVF on sperm motility, SVF (about 13 mg ml^−1^ protein concentration) was diluted with DW and mixed with testicular sperm collected from *C. aeneus*, and sperm movement was observed (Fig. 1F). When testicular sperm were mixed with DW mimicking freshwater, many sperm began to move. Because sperm were hardly detected in the testes of mature male *C. aeneus*, as shown in Fig. 1D, and the motility rate under DW conditions was not very high (approximately 3.5–33.8%), sperm motility was analyzed under mixed conditions with fewer spermatozoa and more testis cells. In the present study, sperm motility in DW was used as the standard value, and sperm motility under other conditions was evaluated relative to this. In Hepes (pH 7.5) buffer, no significant differences in sperm motility were observed compared with control (0.9±0.3). In 1× PBS (100%), the sperm did not move at all (0.0±0.0). However, the use of 0.75× PBS (75%) slightly increased sperm motility to 0.1±0.1, while 0.5× PBS (50%) recovered the sperm motility (0.9±0.8) similar to that in the DW condition (1.0±0.0). These results indicated that the sperm of *C. aeneus* are motile in a hypotonic solution but not in an isotonic solution.

When testicular sperm was treated with SVF, sperm motility was strongly inhibited. The inhibitory effect continued even after SVF was diluted 1000-fold (0.05% SVF). Approximately 10% of the sperm started to move at a 10,000-fold dilution of SVF (0.005% SVF), while approximately 20% were motile at a 100,000-fold dilution (0.0005% SVF).

To determine whether SVF inhibits only the sperm of *C. aeneus* or those of other species as well, testicular sperm from medaka were used for the motility assay (Fig. S1). When testicular sperm were mixed with 154 mmol l^−1^ NaCl saline (control), many sperm began to swim (Yang and Tiersch, 2009). The sperm motility of medaka was strongly decreased to 0.04±0.02 by 5% SVF from *C. aeneus*, whereas 0.5% SVF recovered sperm motility (0.39±0.57) similar to that in the control condition (1±0.0). Additionally, 0.05% SVF completely recovered sperm motility (1±0.07). These results revealed that *C. aeneus* SVF was capable of inhibiting the sperm motility of medaka.

### Identification of SV proteins

**Fig. 2.**
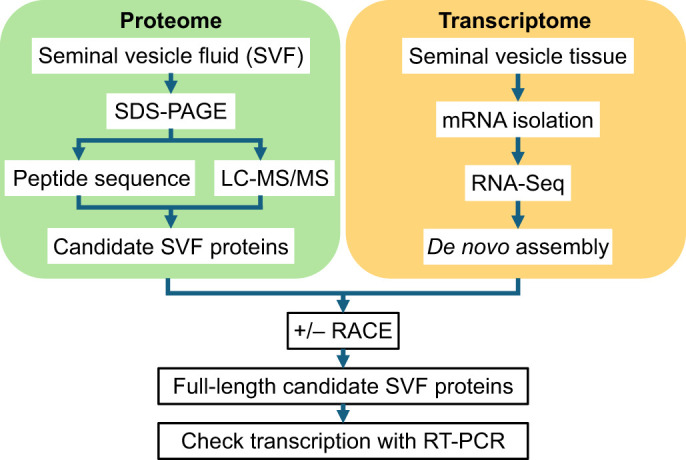
Experimental workflow used to identify the SVF proteins in this study.

**Fig. 3.**
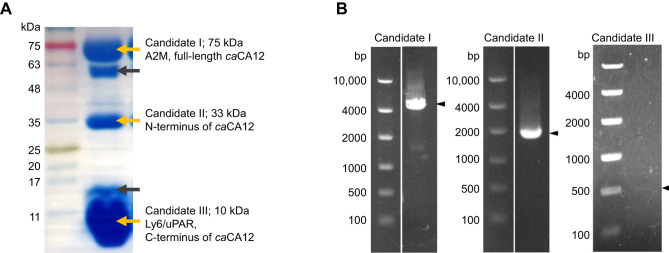
**Identification of proteins in the SVF.** (A) SDS-PAGE of SVF. Yellow arrows indicate bands of each candidate protein with molecular weight and protein names identified. Black arrows indicate bands that could not be identified because of low concentration. (B) Expression of candidate genes I, II and III in SV tissues. Black arrowheads indicate RT-PCR bands of each candidate gene.

To determine the proteins present in the SVF of *C. aeneus*, protein and transcriptome analyses of SV proteins were performed (Fig. 2). In protein analysis, SDS-PAGE of SVF revealed five distinct bands at 75, 60, 33, 15, and 10 kDa (Fig. 3A). The three bands at 75, 33 and 10 kDa represented 21%, 11% and 11% of the total SVF proteins and were designated as candidate molecules I, II and III, respectively. N-Terminal analysis of the three bands was performed by peptide sequencing to identify the proteins in each band. We identified 19 residues (EKTKSFHKKEGKTAGEMHS) in candidate I, 20 residues (APNWSYNGIDGEHQWSDKFP) in candidate II and 20 residues (LQCYDCITNDMTKCIIRQCH) in candidate III.

To obtain more information on the sequences of these proteins, RNA-Seq of SV tissue was performed. Amino acid sequences obtained by peptide sequencing analysis were searched for predictions from the transcriptome data. The results showed that the 19 residues of the candidate I sequence corresponded to amino acids 315–334 of CA12 (Fig. S2A, blue), the 20 residues of candidate II corresponded to amino acids 21–40 of CA12 (Fig. S2A, orange) and the 20 residues of candidate III corresponded to amino acids 20–39 of lymphocyte antigen 6 (Ly6) (Fig. S2B, orange). To confirm the correct full-length sequence of CA12, RACE-PCR was performed and the full-length sequence of candidate II was identified (Fig. S2A).

Despite the different molecular weights of candidates I and II, the amino acid sequences obtained from peptide sequencing of the two bands were partially identical (Fig. S2A). To ascertain whether candidates I and II were the same protein, we performed LC-MS/MS analysis and detected 11 peptides in the candidate I band (Fig. S3A, red characters). The transcriptome data were searched for proteins containing these amino acid sequences, and a candidate containing 1458 amino acids was detected as a match (Fig. S3A). A BLASTp search for this candidate yielded a hit for an alpha-2-macroglobulin (A2M)-like protein from the red bellied piranha *Pygocentrus nattereri* (XP_037391893.1). Based on these results, candidate I was concluded to be *C. aeneus* A2M protein.

Meanwhile, seven peptides were detected in the candidate II band (Fig. S3B). The protein containing the seven peptides was estimated using RNA-Seq to contain 666 bases of the candidate sequence. A BLASTp search with this peptide sequence yielded a hit for CA12 from the Yangtze catfish *Silurus meridionalis* (KAI5100512.1). To verify whether this protein belongs to the CA12 family, a phylogenetic tree of CA protein families in human, mouse and zebrafish was performed (Fig. S4). The phylogenetic tree showed that candidate II protein was indeed a part of the CA12 clade and not in other clades. Therefore, candidate II was considered to be *C. aeneus* CA12 (*ca*CA12).

Candidate III was estimated to be 91–94 amino acids in length and belong to the Ly6/urokinase-type plasminogen activator receptor (uPAR) superfamily (Fig. S2B).

To confirm the gene expression of the three proteins, we performed RT-PCR. Although clear bands were identified for *ca*A2M and *ca*CA12 expression in the SV tissues, only a faint band was detected for candidate III (Fig. 3B).

These results suggested that the SVF of *C. aeneus* contains three proteins, *ca*A2M, *ca*CA12 and a Ly6/uPAR-like protein, which are expressed in the SVs.

### Protein structure of CA12

**Fig. 4.**
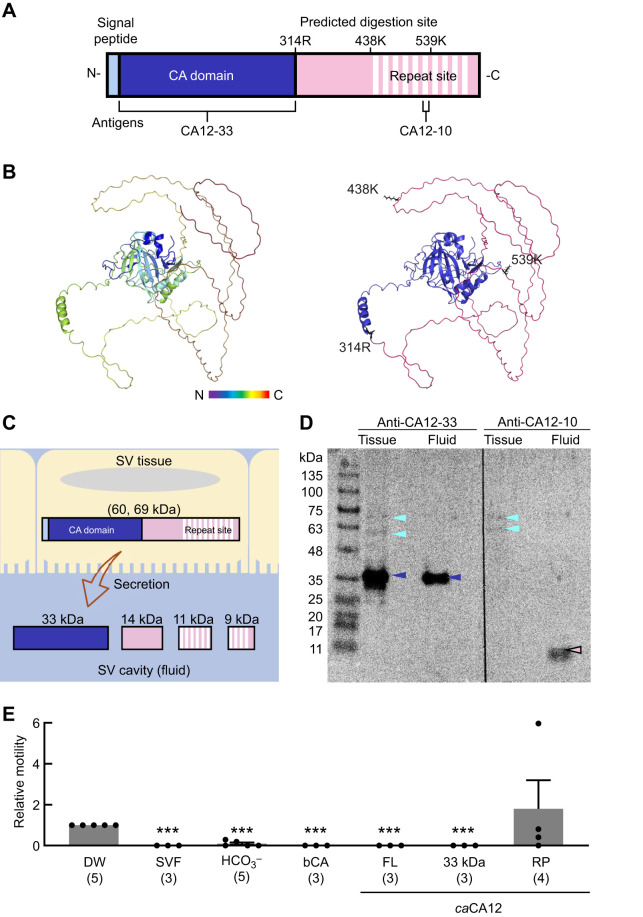
**Structure and function of *C. aeneus* CA12.** (A) 2D structure of *C. aeneus* carbonic anhydrase 12 (*ca*CA12). The three trypsin cleavage sites (314R, 438K and 539K) and regions used for antibody generation (CA12-33 and CA12-10) are indicated. (B) 3D structure of *ca*CA12 as predicted by Alphafold2. Left: color gradient showing the orientation of the protein from the N-terminus (blue) to the C-terminus (red). Right: location of the CA domain (blue); predicted digestion sites (314R, 438 K and 539 K; black); and C-terminal region including the repeated alignment (pink). (C) Schematic diagram of *ca*CA12 secretion from the SV epithelium. (D) Immunoblotting of *ca*CA12 collected from SV tissue (tissue) and SVF (fluid). Two antibodies, anti-*ca*CA12-33 against the CA domain and anti-*ca*CA12-10 against the repeated amino acid domain, were used to detect *ca*CA12. Dark blue arrowheads indicate a band at about 35 kDa corresponding to the CA domain of *ca*CA12. The pink arrowhead indicates a band at about 10 kDa corresponding to the repeat domain of *ca*CA12. Light blue arrowheads indicate bands of about 60 and 70 kDa corresponding to undigested *ca*CA12 in the SV tissue. (E) Motility of *C. aeneus* sperm mixed with SVF, recombinant CA12 proteins or NaHCO_3_ (HCO_3_^−^). Sperm motility was normalized to the number of motile spermatozoa in the control condition using distilled water (DW). Independent experiments were performed using spermatozoa collected from different *C. aeneus* males. All additive proteins were adjusted to 41 µmol l^−1^ as the physiological condition. bCA, bovine CA; FL, full-length *ca*CA12; 33 kDa, N-terminal peptide of *ca*CA domain; RP, repeat domain (TAYPTIQPFAKLPIQP) of *ca*CA12. Data are means+s.d. Numbers in parentheses are number of male *C. aeneus* examined. Unpaired two-tailed Student's *t*-test was conducted (***P<0.001 versus DW).

SDS-PAGE predicted the molecular weight of *ca*CA12 in SVF as 33 kDa. However, the weight of the full-length *ca*CA12 protein predicted from the sequenced cDNA was 69 kDa. Thus, we expected that *ca*CA12 in SVF would be different from full-length *ca*CA12. Alignment of full-length *ca*CA12 showed that it contained a signal peptide and a CA domain at the N-terminus and a repeat alignment of an eight amino acid sequence at residues 441–600 (Fig. 4A). Additionally, we predicted that three sites of *ca*CA12 were digested by trypsin: residues 314R, 438K and 539K in the repeat sequences (Fig. 4A; Fig. S2). Additionally, the conformation of *ca*CA12 predicted by Alphafold2 indicated that the stable CA domain (Fig. 4B, blue) was located in the core, whereas the repeating sequence surrounding the CA domain was unstructured (Fig. 4B, right, pink). The three trypsin cleavage sites in the repeating sequence were exposed on the surface, making it easy for trypsin to access and digest them (Fig. 4B, right).

Owing to the difference in the molecular weight of full-length *ca*CA12 and that predicted by the SDS-PAGE results, we hypothesized that *ca*CA12 was digested to a 33 kDa peptide (containing the CA domain) and other peptides in SV cells (Fig. 4C). To verify this hypothesis, we performed immunoblotting of *ca*CA12. When antibodies against the CA domain (named anti-*ca*CA12-33) were used for immunoblotting, a signals at 33 kDa was detected in both the SV tissue and SVF (Fig. 4D, dark blue arrowheads). Moreover, weak signals of 60 and 70 kDa were observed in the SV tissue (Fig. 4D, light blue arrowheads). Additionally, antibodies against the repeat sequences of *ca*CA12 (anti-*ca*CA12-10) detected a signal of about 10 kDa in the SVF (Fig. 4D, pink arrowhead). Further, weak signals at 60 and 70 kDa were observed in the SV tissue (Fig. 4D, light blue arrowheads), which indicated the presence of undigested *ca*CA12, including the CA domain and repeat sequence. Therefore, during SVF secretion, *ca*CA12 is probably digested into a 33 kDa polypeptide containing the CA domain and an approximately 10 kDa peptide with a repeat domain.

### Effect of *ca*CA12 on sperm motility

To determine whether the digested products of *ca*CA12 inhibit sperm motility, a sperm motility assay was performed for each CA12 peptide at 41 µmol l^−1^, which is the physiological concentration of *ca*CA12 in the SVF. When full-length recombinant *ca*CA12 was mixed with testicular sperm, sperm motility was completely inhibited, as was the case when it was mixed with SVF (Fig. 4E). Additionally, the 33 kDa recombinant protein containing the CA domain also inhibited sperm motility. CA purified from bovine blood also inhibited sperm motility. However, when the recombinant protein of the repeat domain was added to sperm, motility was not inhibited (relative motility 1.8±1.4). Sperm motility in the presence of the repeat domain peptide was 1.8-fold higher than that in DW, although this difference was not significant. Furthermore, in 50 mmol l^−1^ NaHCO_3_, as a mimic of the CA metabolite, sperm motility was significantly decreased. These results indicate that sperm motility of *C. aeneus* is inhibited by CA12 activity in the SVF.

## DISCUSSION

**Fig. 5.**
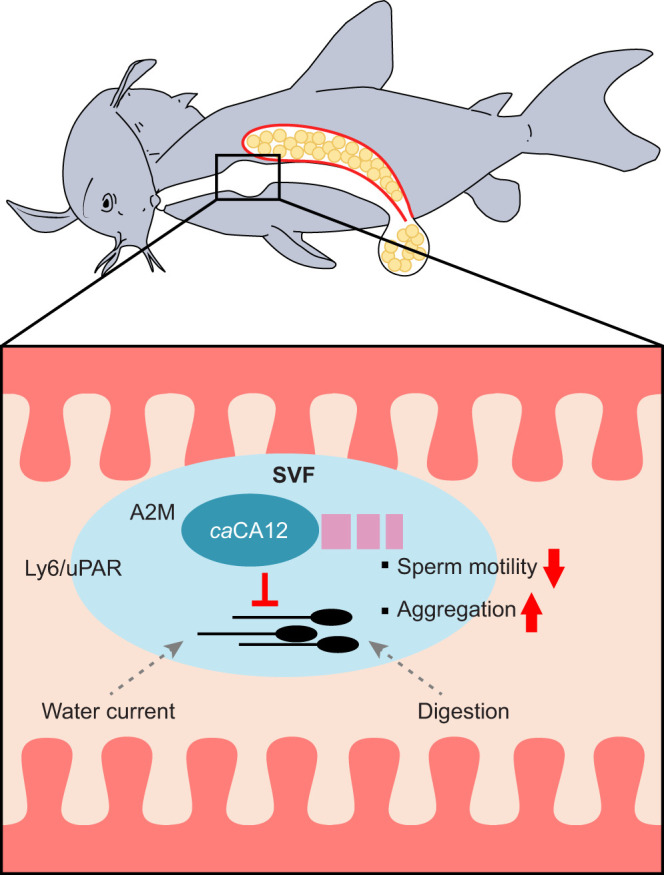
**Mechanism showing the role of SV proteins in sperm drinking.** In SVF, *ca*CA12 inhibits sperm motility immediately after male ejaculation and within the gastrointestinal tract, preventing sperm from expending energy prematurely. The viscosity of SVF, combined with the enzymatic activity of *ca*CA12, acts as a barrier, preventing sperm from diffusing into the gastrointestinal tract and so protecting sperm from digestion. After passing through the gastrointestinal tract, *ca*CA12 is digested or removed from the sperm surface, allowing sperm to regain motility and move towards the eggs. A2M, alpha-2-macroglobulin; Ly6/uPAR, lymphocyte antigen 6/urokinase-type plasminogen activator receptor.

In this study, we identified three proteins, *ca*CA12, *ca*A2M and a Ly6/uPAR-like protein, that constitute the SVF in *C. aeneus* (Fig. 5). SVF and *ca*CA12 serve to suppress sperm motility. *Corydoras aeneus* SVF inhibited not only the motility of *C. aeneus* sperm but also that of medaka sperm. These results suggest that SVF is designed to suppress sperm motility during passage through the digestive tract and allows sperm to initiate movement only when they are close to the egg. Thus, the energy conservation enables sperm to begin to move in the right place for the successful reproductive mode of sperm drinking in *C. aeneus*.

*ca*CA12 contains a unique repeat sequence at its C-terminus (Fig. 4). Our results indicate that *ca*CA12 is digested into two parts when secreted from the SV epithelium: a 33 kDa CA domain and an approximately 10 kDa repeat sequence. Inhibition of sperm motility was observed in the presence of the CA domain but not with the repeat sequence (Fig. 4E). These results suggest that the sperm-inhibitory effect of SVF can be attributed to the CA domain of *ca*CA12. Generally, CA catalyzes the interconversion of CO_2_ and HCO_3_^−^ and is involved in many physiological processes such as pH regulation, CO_2_/HCO_3_^−^ transport and water/electrolyte balance (Sly and Hu, 1995). In our study, NaHCO_3_ inhibited *C. aeneus* sperm motility (Fig. 4E). Flatfish sperm contain a large CA, and sperm flagellar movement is arrested in the presence of NaHCO₃ and CO_2_ gas (Inaba et al., 2003). Flagellar CA probably converts intracellular HCO_3_^−^ to extracellular CO_2_, increasing intracellular pH and thereby initiating sperm motility. In the case of *C. aeneus* SVF, CA is present outside the sperm, and *ca*CA12 may increase the CO_2_ concentration and decrease the extracellular pH, resulting in the arrest of flagellar movement. An alkaline environment and low CO_2_ concentrations have been reported to be important for sperm motility in rainbow trout (Woolsey et al., 2006).

In the present study, the SVF of *C. aeneus* was characterized by both viscosity, due to the high protein concentration, and *ca*CA12 enzymatic activity (Figs 1, 4 and 5). Also, *C. aeneus* SVF and bovine CA inhibited the motility of medaka sperm (Fig. S1). These results suggested a similar mechanism of sperm flagella regulation in teleosts. Sperm flagellar motility is strictly regulated, as spermatozoa are monocellular and have difficulty obtaining energy to keep moving. In particular, the motile period of fish sperm, such as that of halibut and turbot sperm, is extremely short compared with that of mammalian sperm (Cosson et al., 2008). For efficient fertilization, the mechanism of sperm motility initiation and the duration of motility must be regulated; transient inhibition by seminal plasma is especially common across species. The most prominent protein in human semen is semenogelin I/II, which physically gelatinizes the semen, delays the onset of sperm motility and helps sperm survive in the dangerous vaginal environment (Yoshida et al., 2008). It is interesting that a phenomenon similar to that observed in human semen is observed during sperm drinking in *C. aeneus*.

In the present study, we found that *ca*CA12 protein was secreted into the SVF (Figs 3A and 4D), unlike mammalian CA12, which is typically membrane bound because of its transmembrane domain (Gilmour and Perry, 2009). Comparative analysis of CA proteins in mouse, human and zebrafish revealed that the subcellular localization was basically identical for each CA protein (Fig. S4). However, zebrafish CA12 exhibited a distinct localization pattern compared with its mammalian counterparts, specifically lacking the transmembrane domain and displaying a secretory phenotype. Notably, *ca*CA12 and zebrafish CA12 shared a common feature: loss of the transmembrane domain at the C-terminus. These results suggest that CA12 lost its transmembrane domain in the common ancestor of *Corydoras* and zebrafish. Secreted CA12 may play a unique role in otophysan fish, warranting further investigation to elucidate its mechanism.

In terms of peptide sequence, *ca*CA12 was detected in both the 75 and 33 kDa bands (Fig. S2A). However, LC-MS/MS of the 75 kDa band detected 11 peptides, which were included in A2M (Fig. S3A). Transcriptome data predicted that full-length *ca*CA12 was 69 kDa (Fig. S3A) and that A2M has a similar molecular size of 75 kDa (Fig. 3). Taken together, these results suggested that the 75 kDa band of candidate I might include both *ca*A2M and the full-length *ca*CA12, and that peptide sequence analysis might detect a part of *ca*CA12. Moreover, it was difficult to verify the function of A2M because *C. aeneus* A2M recombinant protein could not be produced or purified. It has recently been reported that A2M functions as a broad-spectrum protease inhibitor under extracellular conditions (Vandooren and Itoh, 2021). This led us to hypothesize that *ca*A2M as a protease inhibitor in SVF contributes to sperm survival in sperm drinking by inhibiting sperm digestion in the female gastrointestinal tract. However, it is surprising that caCA12 in SVF was digested from 69 kDa to 33 kDa and ∼10 kDa despite of the presence of protease inhibitor A2M. It is contradictory to observe both protease inhibition and protein cleavage occurring simultaneously. Although the mechanism by which these proteins are made in seminal vesicles is not entirely clear, there may be differences in expression of cell types and tissues between *ca*CA12 and *ca*A2M.

Based on peptide sequencing and RNA-Seq, the 10 kDa band was predicted to be a protein that belongs to the Ly6/uPAR family. As shown in Fig. 3B, RT-PCR of the SV tissue failed to produce a clear band, which led us to conclude that the protein remained unidentified; proteins belonging to the Ly6/uPAR superfamily have highly conserved cysteine sequences but not other amino acid sequences (Guo et al., 2015; Herberg et al., 2018; Hijazi et al., 2009; Loughner et al., 2016), making it difficult to annotate them using only mRNA and fragmentary amino acid sequences. Moreover, the C-terminal end of *ca*CA12 was found to be degraded into an approximately 10 kDa peptide. Collectively, these results indicate that the large 10 kDa band is presumably composed of multiple proteins, including the Ly6/uPAR family protein and the C-terminal peptide of *ca*CA12.

Despite the challenges in full identification, candidate III was conclusively categorized as a Ly6 family member. Generally, there are two types of Ly6/uPAR proteins: secreted and glycosylphosphatidylinositol (GPI)-anchored types. The 10 kDa protein in SVF was thought to be the secreted type. A previous study showed that mouse SV secretions contain a secreted Ly6/uPAR protein, PATE4 (SVS7), with multiple functions, including (1) a calcium transfer inhibitor (Coronel et al., 1992), (2) an enhancer of sperm motility (Luo et al., 2001) and (3) an antibacterial protein (Morohoshi et al., 2021). Based on these collective findings, we hypothesized that the candidate III protein in SVF may function as a manipulator of sperm motility or an antibacterial protein in sperm drinking, but its function was not investigated in this study. Further studies are required to identify the candidate III protein and analyze its functions.

Histochemical staining of SV from *C. aeneus* showed that SVF was positive for eosin, indicating the presence of eosinophilic contents (Fig. 1E). A previous study reported that the SV contents of *C. aeneus* are periodic acid–Schiff (PAS) positive (Franceschini-Vicentini et al., 2007), implying that the SV contents of *C. aeneus* are acidophilic and contain glycoproteins. Other studies in mammals have also reported that SV contents are positive for eosin (Creasy et al., 2012; Sayed et al., 2014; Welsh et al., 2010). Although mammals and *Corydoras* independently acquired SVs, the material properties of SV contents may be similar between these two groups. There is a previous report on CA activity in human semen (Mawson and Fischer, 1953). The effect of CA on sperm motility in human semen is unknown, but our results suggest the existence of a common rule by which animals acquired SVF traits during the evolutionary process.

## Supplementary Material

10.1242/jexbio.250293_sup1 Supplementary information
