# Supplementary material for: Seminal vesicle protein caCA12 in Corydoras aeneus inhibits sperm motility for sperm drinking
Source: J Exp Biol. 2025 May 15;228(9):jeb250293. doi: 10.1242/jeb.250293 (PMC12091868; doi:10.1242/jeb.250293)
Supplement: Supplementary information [file jexbio-228-250293-s1.pdf]

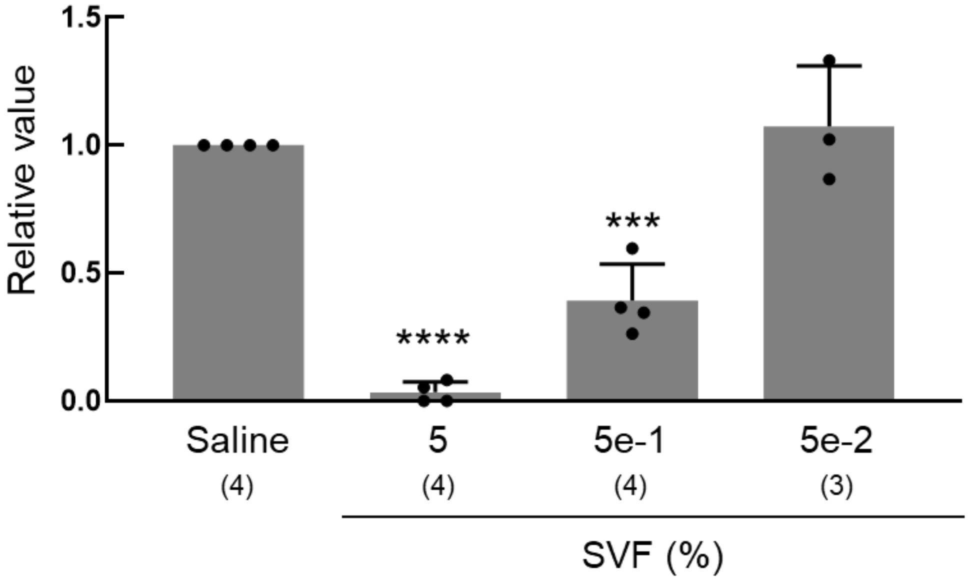

Fig. S1. Effect of *Corydoras* SVF on the motility of other species' sperm.

Medaka (*Oryzias latipes*) sperm was diluted with saline or SVF. Sperm motility was normalized to the number of motile spermatozoa in the saline control condition (control). Independent experiments were performed using spermatozoa collected from different male medaka. 50% SVF/DW was diluted to 0.1-fold (5%), 0.01-fold (5e-1%), and 0.001-fold (5e-2%). Unpaired two-tailed Student's *t*-test was conducted, ***$P < 0.001$, ****$P < 0.0001$ vs. Saline.

Journal of Experimental Biology • Supplementary information

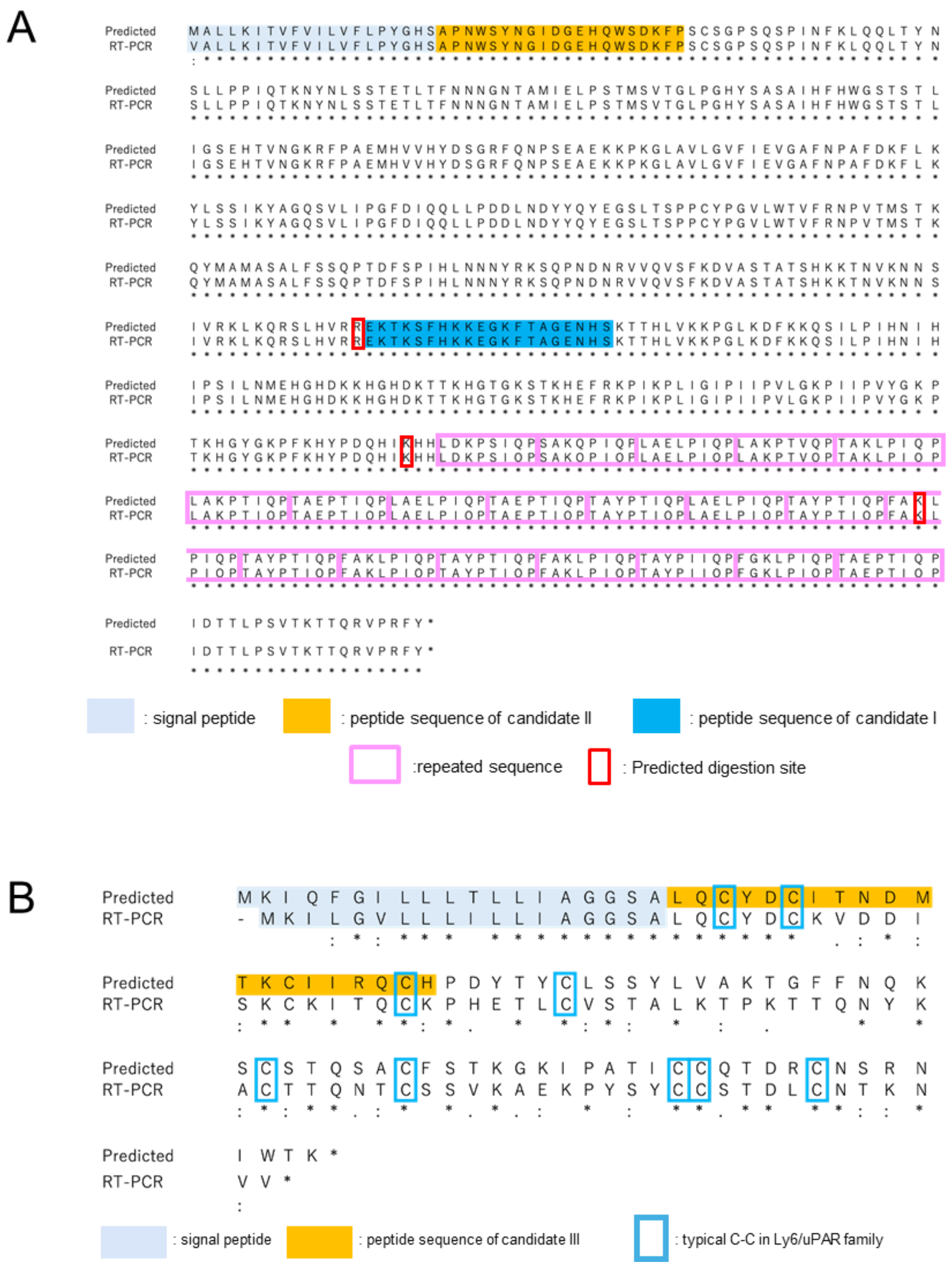

Fig. S2. Amino acid alignments of candidate II and III proteins.

(A) The top alignment was predicted by transcriptome analysis, and the bottom alignment was predicted from the results of DNA sequencing using RT-PCR. The gray marker indicates the signal peptide, orange marker indicates the matching part of the transcriptome result and

Journal of Experimental Biology • Supplementary information

the peptide sequence of the candidate II band, and blue marker indicates the matching part of the transcriptome result and the peptide sequence of the candidate I band. Red plates indicate the predicted trypsin digestion sites. The pink plates indicate the repeated sequences. (B) The top alignment was predicted by transcriptome analysis, and the bottom alignment was predicted from the results of DNA sequencing using RT-PCR. The gray marker indicates the signal peptide and the orange marker indicates the matching part of the transcriptome result and the peptide sequence of candidate band III. Blue plates indicate the typical disulfide binding sites of Ly6/uPAR family proteins.

A

1 MCSRMKLLSGIPAVLLLLHI

21 AAAQKLANDTIYLVTLNSQP

41 VGGSTETLCVHVNPLRPIFS

61 LLVNLKFGSTRQTLLTERFI

81 NMEYYQCKQFQVPEVRAETE

101 ASVTVLISGPKTSFNKTSMI

121 LIKPGSEMVIMQTDKPIYKP

141 GQTVKFRIASLYPSFLTYNQ

161 MFPTIELQDPNGNRIGQWLN

181 SSTGNGLIDLLYPTNPESPL

201 GFYVITAWNKNNDVFTQTFE

221 LKDYVLPKFEVTVKLPDVIT

241 IVDASATLNICAKYTYGKPV

261 SGSVKATVCRNKYPWLREET

281 TDKDICIKFTQTTDATGCIS

301 RVLDLTKFSLTKTNYEDMIQ

321 VTCDVEEFATGIIISGSSAV

341 YVTSELIRLTFENSASVFKV

361 GMNFDGVVKAEDQNSKPLIN

381 RLLYLKITYGDNAVSERTLT

401 TDINGLAKFSLDTQMWGNSS

421 VTLQARYYKSEKQPPYDPNV

441 RLPIIPQAYLWLQSFISNSN

461 SFLTLVPSADPFSCEQVATV

481 TAKYLIHSSTLRASQQSLPI

501 FYMVMSKGRVVQQGRLIIYV

521 NARREENRGTVTLTLNSMKT

541 LPPVAQVLLYAILPSGEAIA

561 DSMNFPIENCLPNKVSLSFS

581 SAQELPAGKTKLTLKAQPGS

601 LCSVRAIDQSLLLLQPEKEL

621 NAEAVFSLLPVQVLSGYPYN

641 IDDERTYCVDTPPVDPLPVP

661 ILLPRVRRSKFFFPYGSQSD

681 VYGVFKNMAMKILTNADIKK

701 PMSCYYGPFWDVRREPGVVF

721 NFAVAKESSVSGASAPEQPV

741 TTVRKFFPATWIWDLVPVGD

761 SGMMAVDETLPDTITKWQAG

781 AFCTSSVGFGVAPKVELTAF

801 QPFFVSLTLPSSVIRKEMFT

821 LKATVFNYLQGCMAVNVDLA

841 PSPLFVARPCKGCIYSSCLC

861 ADQSYTFSWIITANVVGEAS

881 INVTAAAVQSSTLCGRNDIT

901 VPQKGRIDTVINTLLVQAEG

921 TKQTTSYNELICSSGGAVEV

941 PVSLSLPELYVEGSVTAWVS

961 VLGDIMGRALNNLASLLQMP

981 YGCGEQNMLLFAPNIYILRY

1001 LESSGQLTEAIRSKAETFLI

1021 GGYQRELTYKHDDGSYSAFG

1041 MSDKSGNTWLTAFVMKAFAG

1061 AKRYIFIDDLYINQARIWLG

1081 QQQQENGCFASVGQLFHTDM

1101 KGGVDDEVTLTAYIMSAMLE

1121 LGLNLTDPVVGKALKCIRSA

1141 TPQLTSTYALALLSYTFTLA

1161 GDQSSRSSVISKLNSIAIIS

1181 DGTRHWSRQNTGTVDSLEVE

1201 MTSYVLLTLLTGPTLPGYDI

1221 SYTSSIVRWLAKQQNAFGGF

1241 ASTQDTVVALEALAKYSAAT

1261 YRPSGSMAVRVTSPLGKTKD

1281 FTVNQSNRLLYQESALQEVQ

1301 GNYKVKATGSGCVYVQFTLH

1321 YNIPPPADDSSFSIKASTKG

1341 NCSIPIPTVQVTVTVRFNGN

1361 RKKTNMMVIDLKPLSGFSLV

1381 SDSVLKVTESSDGNVKRVDQ

1401 KDGHVIVYLDYLIQGADRTY

1421 TLVIQQDVAVQNLQPAVVKV

1441 YDYYETVAEAVTEYTSPC

B

1 MALLKITVFVILVFLPYGHS

21 APNWSYNGIDGEHQWSDKFP

41 SCSGPSQSPINFKLQQLTYN

61 SLLPPIQTKNYNLSSTETLT

81 FNNNGNTAMIELPSTMSVTG

101 LPGHYSASAIHFHWGSTSTL

121 IGSEHTVNGKRFPAEMHVVH

141 YDSGRFQNPSEAEKKPKGLA

161 VLGVFIEVGAFNPAFDKFLK

181 YLSSIKYAGQSVLIPGFDIQ

201 QLLPDDLNDYYQYEGSLTSP

221 PCYPGVLWTVFRNPVTMSTK

241 QYMAMASALFSSQPTDFSPI

261 HLNNNYRKSQPNDNRVVQVS

281 FKDVASTATSHKKTNVKNNS

301 IVRKLKQRSLHVRREKTKSF

321 HKKEGKFTAGENHSKTTHLV

341 KKPGLKDFKKQSILPIHNIH

361 IPSILNMEHGHDKKHGHDKT

381 TKHGTGKSTKHEFRKPIKPL

401 IGIPIIPVLGKPIIPVYGKP

421 TKHGYGKPFKHYPDQHIKHH

441 LDKPSIQPSAKQPIQPLAEL

461 PIQPLAKPTVQPTAKLPIQP

481 LAKPTIQPTAEPTIQPLAEL

501 PIQPTAEPTIQPTAYPTIQP

521 LAELPIQPTAYPTIQPFAKL

541 PIQPTAYPTIQPFAKLPIQP

561 TAYPTIQPFAKLPIQPTAYP

581 IIQPFGKLPIQPTAEPTIQP

601 IDTTLPSVTKTTQRVPRFY

Fig. S3. The amino acid sequences of candidate I and II that matched with the transcriptome data from LC-MS/MS.

(A) The amino acid sequence was predicted as A2M from the transcriptome results, and the parts of red sequence were matched with the LC-MS/MS result of candidate I. (B) The amino acid sequence was predicted as CA12 from the transcriptome results, and the parts of the red sequence were matched with the LC-MS/MS result of candidate II.

Journal of Experimental Biology • Supplementary information

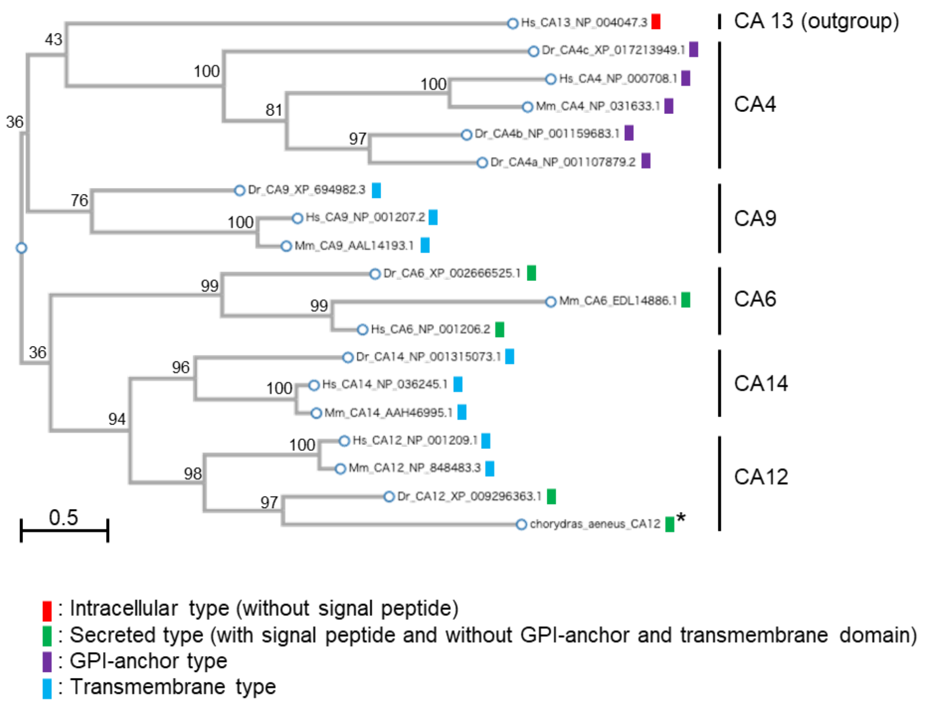

Fig. S4. The maximum-likelihood tree of carbonic anhydrase

Maximum likelihood tree showing the relationship between 19 samples of extracellular-type CA proteins (CA4, 6, 9, 12, and 14) of human, mouse, and zebrafish plus one outgroup intracellular-type CA protein (human CA13). Numerical values represent the bootstrap values (out of 100). Scale shows substitutions per site. Asterisk represents *ca*CA12 sequences obtained in this study.

Journal of Experimental Biology • Supplementary information

Table S1. Settings for sperm motility analysis and PCR primers used in this study.

Table S1A. Parameters for SMAS analysis of *C. aeneus* sperm.

Data processing	min.	max.
Binarization	150	255
Deletion of particle out of edge	0	0
Deletion of particles (area [pixel])	50	250
Deletion of particles (roundness)	0.9	1.5
Deletion of particles (minimum center of gravity distance/ average center of gravity distance)	0	1
Deletion of particles (Oblateness)	1	2

Table S1B. Parameters for SMAS analysis of medaka sperm.

Data processing	min.	max.
Binarization	150	255
Deletion of particle out of edge	0	0
Deletion of particles (area [pixel])	10	39
Deletion of particles (roundness)	0.9	1.5
Deletion of particles (minimum center of gravity distance/ average center of gravity distance)	0	1
Deletion of particles (Oblateness)	1	2

Table S1C. Primers for PCR.

Primer ID	Sequence (5′→3′)
GeneRacer™ 5′Primer	CGACTGGAGCACGAGGACACTGA
Reverse GSP-1	AAGTTTATTGGAGACTGGGATGGT
Forward GSP	CGAGGACACTGACATGGACTG
GeneRacerTM 3′ Primer	CGTTACGTAGCGTATCGTTGACAGC
GeneRacerTM 5′ Nested Primer	GGACACTGACATGGACTGAAGGAGTA
Reverse Nested GSP	TCTGCTCCGAACTGGTCCTA
Forward Nested GSP	CACCAGTGGTCGGACAAGTT
GeneRacerTM 3′ Nested Primer	CGTTACGTAGCGTATCGTTGACAGC
T7 promotor Primer	TAATACGACTCACTATAGGG
T3 promotor Primer	ATTAACCCTCACTAAAGGGA
forward Primer for cloning candidate I	ATGAAGCTCCTCAGTGGGATTC
Reverse Primer for cloning candidate I	TTAACATGGGGAGGTGTATTCA
forward Primer for cloning candidate II	ATGGCACTCCTCAAAATAACAG
Reverse Primer for cloning candidate II	ATAAAATCGAGGGACTCGCTG
forward Primer for cloning candidate III	GCATCCACCCACTTCTTCCT
Reverse Primer for cloning candidate III	TTGAACAGTCAGATGCTAATGCA
Forward Primer for subcloning caCA12-33	CCCGAATTCTGGTCGGACAAGTTCCCAT
Reverse Primer for subcloning caCA12-33	GGGCTCGAGGGTAGCAGGGAGGTGAGGTA
Forward Primer for subcloning caCA12-10	CCCCCATGGAGAAGACCAAAAGCTTCCAC
Reverse Primer for subcloning caCA12-10	CTCGAGATAAAATCGAGGGACTCGCTG
pGEX_5_Primer	GGGCTGGCAAGCCACGTTTGGTG
pGEX_3_Primer	CCGGGAGCTGCATGTGTCAGAGG
Forward Primer for subcloning caCA12-FL	CCCGAATTCGCTCCGAACTGGTCCTATAA
Reverse Primer for subcloning caCA12-FL	GGGCTCGAGATAAAATCGAGGGACTCGCTG
T7 terminator Primer	ATGCTAGTTATTGCTCAGCGG

Journal of Experimental Biology • Supplementary information
